# ‘In God We Trust’: The role of religion in COVID-19 vaccinations in Dar es Salaam, Tanzania

**DOI:** 10.4102/jphia.v16i3.707

**Published:** 2025-04-18

**Authors:** Thomas J. Ndaluka, Ambrose T. Kessy, Chima E. Onuekwe

**Affiliations:** 1 Department of Sociology and Anthropology, Faculty of Social Sciences, University of Dar es Salaam, Dar es Salaam, United Republic of Tanzania; 2 Directorate of Research, Publications and Consultancy, The University of Dodoma, Dodoma, United Republic of Tanzania; 3 Planning, Finance and Administration, Dar es Salaam, United Republic of Tanzania; 4 Department of Immunizations, Emergency Preparedness and Response (EPR), World Health Organization, Dar es Salaam, United Republic of Tanzania; 5 Centre for Health and Allied Legal and Demographical Development, Research and Training (CHALADDRAT), World Health Organization, Awka, Nigeria

**Keywords:** COVID-19 vaccines, Pentecostal believers, COVID-19, socio-ecological model, vaccine rejection, believers’ attitude, Tanzania

## Abstract

**Background:**

After the outbreak of COVID-19, the World Health Organization (WHO) identified vaccines as one of the intervention mechanisms capable of controlling and preventing COVID-19 infections. However, the uptake of the vaccine was below the expectation, while the cause for such manifestation was unclear.

**Aim:**

This study aimed to examine the attitude of Pentecostal believers towards COVID-19 vaccines in Dar es Salaam, Tanzania. The focus was to investigate the role of religion in COVID-19 vaccinations.

**Setting:**

This study was conducted at three Pentecostal churches, namely Arise and Shine Ministry, Ufufuo na Uzima Ministry and Tanzania Assemblies of God – Makongo-juu, all located in Dar es Salaam, Tanzania.

**Methods:**

The study employed qualitative interviews to generate information from 55 Pentecostal believers.

**Results:**

Findings from this study attest that the attitude of Pentecostal believers towards COVID-19 vaccines was mixed; some hesitated to be vaccinated and another quarter accepted vaccination. Despite being provided free of charge, the uptake of the COVID-19 vaccine was attributed to the social-ecological factors that the individuals were in.

**Conclusion:**

Religion has remained a key factor for hesitancy toward COVID-19 vaccines among believers. The best way to increase acceptance of COVID-19 among believers, is to have an appreciation of the socio-cultural and ecological environment where Individuals’ member resources are stored. Acceptance of COVID- 19 was not only related to scientific and medical factors, but rather religious issue as well.

**Contribution:**

It contributes to public health efforts that acknowledges the engagement of religious and socio-cultural dimensions to disease outbreaks and interventions.

## Introduction

The World Health Organization (WHO) treats vaccines as the most effective, public health intervention for preventing and controlling infectious disease outbreaks including COVID-19.^1^ Indeed, the WHO identified the vaccine as one of the intervention mechanisms capable of controlling and preventing further COVID-19.^2,3^ It was confirmed that COVID-19 vaccines can provide much-needed immunity to COVID-19.^2,3^ Consequently, the frantic efforts aimed to control the spread of the virus led to the introduction of several types of vaccines, with some still undergoing clinical trials to determine their efficacy. By the end of 2021, COVID-19 vaccines were progressively distributed to nearly all countries, with about one billion doses distributed through the COVID-19 Vaccines Global Access, abbreviated as COVAX.^2^ Since then, the Tanzania government instituted serious measures aimed at controlling and preventing the spread of the virus.^4,5^ For instance, it approved six COVID-19 vaccines to be used in the country. These are Janssen (Johnson & Johnson) made in the United States (US), Moderna (made in the US), Gamaleya (made in Russia), Pfizer/BioNTech (made in Germany) and Sinopharm (made in China).^3^ The ANCoV 1.3.20, a Tanzanian-made vaccine was still under clinical trial.^3^

Despite the efforts by the Tanzania government and international organisations as explained above, several factors have undermined COVID-19 vaccination efforts. These include individualised fears associated with the potential effects including loss of fertility, insertion of microchips into their bodies for monitoring purposes (based on unfounded rumours) and more significantly and on a seemingly larger scale because of the collective nature of such rhetoric – religious beliefs and intervention.^6^ Certain individuals expressed fears that COVID-19 vaccines could affect their immune system. Other factors were attributed to the lack of transport costs to vaccination service and the lack of time because of childcare and work and fears about death after vaccination.^6^ Other studies mentioned demographic characteristics such as age, gender, education level, profession, ethnicity, race, religion and income level as the determinants for rejection of COVID-19 vaccination.^6,7,8,9^ Older people were more likely to get vaccinated than younger people as they are more vulnerable to virus attacks than their younger counterparts.^6^ Moreover, women were also more likely to be vaccinated than men.^9^ Furthermore, a higher level of education is linked to being amenable to vaccination and so are individuals who work in health professions.^6^ Workers who travel abroad are also more likely to be vaccinated than those who are unemployed or employed in the informal sector, which does not encourage travelling to other countries. Studies also show that businessmen and women were more likely to accept vaccination because of travel conditions and restrictions imposed on unvaccinated travellers.^10,11^

What the above-mentioned studies, including that by Hoare et al., did not embrace is the role of religion and religious and political leaders in either positively or negatively influencing the uptake of COVID-19 vaccinations.^6^ In this regard, religious leaders and fellow believers with a positive attitude towards COVID-19 vaccination can encourage their followers to accept vaccinations. Schimitzberger et al. identified four reasons for hesitance: political ideology, religious objections, conspiracy theories and misinformation.^12^ Bogart et al. added transparency as a reason capable of boosting acceptance of COVID-19 vaccines.^13^ This study attested that transparent information about COVID-19 vaccine efficacy and safety could enhance acceptance and consequently reduce hesitancy.^13^

As for religious grounds, tight-knit religious communities resisted vaccinations just as poor people were more likely to reject vaccination than wealthier ones.^12,13,14^ Unlike other Christian denominations such as the Catholic and Lutheran churches, Pentecostal believers were considered to be one of the tight-knit religious communities, and thus the Pentecostal theology provides an alternative moral world view about their health and community.^15^ More often, Pentecostal churches have been unwilling to reason with scientific explanations and strategies about COVID-19.^16^ In this vein, Cantarero underscored the objections of Pentecostals in the United States against COVID-19 vaccines and strategies.^16^ Guidry et al.^17^ also observed that Evangelical Christians had a negative attitude towards COVID-19 vaccines. Nevertheless, believers who sought advice from their religious leaders were reported to be more hesitant than those who perceived the benefits of having the COVID-19 vaccine.^16^ According to Cantarero^18^, Pentecostals used different means to show their objections towards COVID-19 vaccination strategies, namely public statements, legal action and other public activities.^16^

This study seeks to understand whether the position of the churches as advanced by Cantarero and others had impacts on COVID-19 vaccinations in Tanzania.^16^ Our main questions are: (1) *What role does a person’s religion play in his attitude, perception and acceptance of COVID-19 vaccinations*? (2) *Does Pentecostal belief induce less health-seeking behaviour and consequently rejection of COVID-19 vaccines*? (3) *Does the position of religious leaders affect the attitude of their followers about COVID-19 vaccinations*? This study intends to examine the position of the Pentecostal churches and whether that affected the perception, attitude and acceptance among their believers.

The socio-ecological model assumes that individuals are predisposed to several conditions that come from their surrounding environment. The socio-ecological model places the individual at the centre of the circles. Urie Bronfenbrener in the 1970s proposed five levels in the circles: the micro-system, meso-system, exo-system, macrosystem and chrono-system. This study focused on the first three systems, namely micro-system, meso-system and exo-system. The micro-system level includes an individual’s immediate environment such as his or her family members, relatives, schoolmates and friends, and the workplace where he or she meets with co-workers, managers and directors. On the other hand, the meso-system, which is the second level, comprises the social system that includes institutions and religious organisations such as the Pentecostal churches where the individual is exposed to the teaching and orientation of a particular religion. Finally, the exo-system comprises formal and informal structures such as social networks, the media and government policy.^8^ Significantly, the Bronfendbrener model has been revised and simplified to focus on four dimensions: (1) individual, (2) community (i.e., the socio-cultural), (3) institution (including an organisation) and (4) ecological and policy.

At the individual and interpersonal (micro) system, the analysis focuses on gender, age, income status, education level, knowledge, attitudes, personality and previous experience.^8,19,20^ The individual level focuses on individual attributes, qualities and attitudes towards those who are in direct contact with him or her. Those closer to the individual can include family members, religious fellows, religious leaders, friends and peers, workplace members, neighbourhood and business partners.^20^ Under the socio-ecological model, an individual is an outcome of interpersonal inspiration. In this regard, a person whose close religious fellows were vaccinated or have a positive attitude towards vaccines would have a positive attitude to COVID-19 vaccines. Conversely, when religious members, their religious leaders and close friends of a person have a negative attitude for COVID-19, it can reduce the chances of that person getting vaccinated. Implicitly, an individual’s choice to either get the COVID-19 vaccine or not would also depend on the type of religious members and close peers.

As religious beliefs and religion are important institutions in the community, they also play a key role in imparting religious values and reciprocity among religious believers.^8^ Usually, devout adherents tend to uphold certain values identifiable with their religious denomination, including how to behave, wear clothing, eat (diet) foods and even speak in public. In many cases, believers tend to mimic the behaviour (demeanour) of their religious leaders, which in this case also includes health-seeking behaviour. For instance, if the leader insists on religious healing versus medical prognosis, many of the adherents could also desist from seeking such medical care. In Tanzania as elsewhere, many religious institutions are responsible for providing non-religious services such as education, health and psycho-social support. In general, the analysis at this level focuses on various community-based services, opportunities and choices available to the faithful of a given religious grouping.

Finally, the ecological and policy level is not only about weather conditions, terrain, natural endowment, seasonality and geographical location but also about policy factors that inspire an individual’s access to services.^8,21^ These factors include government policies, strategies, laws, regulations and priorities.^8,20,21^ In general, at this level, the focus is based on public policies on COVID-19 vaccine uptake and government priorities in delivering essential services to the communities. In this regard, the Tanzania government has enacted several laws, introduced policies and provided regulations including free access to COVID-19 vaccines aimed to encourage Tanzanians to get vaccinated against the lethal virus.

## Research methods and design

### Data collection

The data in this article were generated in January 2022 using a case study research design. As established by Guidry et al., religion played a role in believers’ attitudes towards COVID-19 hesitancy and acceptance in the US.^17^ However, unlike their study, this study is limited to three Pentecostal churches as case studies, namely the Tanzania Assemblies of God, commonly known as TAG (Makongo), Ufufuo na Uzima (literal translation, life and resurrection) and Arise and Shine Ministry. The selection of these three churches was purposive. The believers of these churches possessed valuable information needed to answer the research question. Moreover, most studies focus on the mainstream churches such as the Catholic, Lutheran and other protestant denominations. The Pentecostal churches are often side-lined making their voices marginalised. This study intends to gain insight from this so-considered tight-knight society on the role of religion in COVID-19 vaccination in Tanzania.

In-depth interviews were conducted in the Kiswahili language, which is known by all interlocutors. The extracts were translated into English to enable a wider understanding to the readers who are not vested in Kiswahili. In order to maintain the information from interlocutors and to ensure that the meaning was not lost as a result of translation into the English language, the authors ensured to remain as close to the original Kiswahili version as possible. The interviews were conducted by the authors who were assisted by six research assistants. The interviews enabled the generation of confidential information that would otherwise be difficult to obtain using other methods. A total of 55 participants were interviewed, that is, 27 from TAG – Makongo-Juu, 16 from Ufufuo na Uzima and 12 from the Arise and Shine Ministries. Table 1 summarises the number of interviewed individuals per denomination.

**TABLE 1:** Number of interviewed individuals per Pentecostal church.

Variable	Denomination			
	Arise and Shine Ministries	TAG – Makongo-Juu	Ufufuo na Uzima Ministry	Total
**Number of interlocutors by gender**				
Female	7	11	7	25
Male	5	15	9	29
Total	12	26	16	54
**Education level of interlocutors**				
Primary	1	6	4	11
O-level	5	8	6	19
A-level	1	2	1	4
Tertiary	5	10	5	20
**Average age (years)**				
18–24	2	4	1	7
25–35	8	11	7	26
36–45	1	7	8	16
46 >	1	4	0	5

TAG, Tanzania Assemblies of God.

The participants were selected purposively because they had the lived experience and also possessed information, which narrated their attitude and perception about COVID-19 vaccines. The purposive sampling technique was preferred because the study desired to have people who had the experience and willingness to share their perceptions on COVID-19 vaccines. After obtaining permission from the religious leader, the research team purposely approached believers of the denomination for their consent to be interviewed. Participation in this study was voluntary. The criteria for being selected were age, gender and membership to the denomination. The sample size was not predetermined but was achieved after gaining a saturation point, and thus, the information obtained from the interviews was sufficient to provide the narrative of believers towards COVID-19 vaccines. An interview guide was used for questioning so that similar questions could be asked to all participants.

The study subjected the resulting qualitative information to thematic analysis. Data analysis started during fieldwork. Discussions went together with reflection such that some issues for discussion were raised when reflected on what was said continually (researchers engaged with interlocutors). This was necessary for the joint creation of information or reality. Furthermore, the analysis entailed sieving through the detailed information collected from the field before establishing patterns corresponding with certain themes with the objective of the study. The findings are largely presented in narrative form with the voice of the participants presented verbatim as short extracts presenting evidential testimonies coupled with a reflective interpretation of the narrative.

### Ethical considerations

Ethical clearance to conduct this study was obtained from the University of Dar Es Salaam (UDSM) (No. MPEC/R/10/1).

This study was conducted under the aegis of the UDSM research policy and operational procedures, and UDSM Research Ethics Policy (www.udsm.ac.tz). Participants who were involved in this study provided informed consent before taking part. Participation in this study was voluntary.

In line with the research protocol, the study considered all applicable ethical issues, including obtaining permission from relevant authorities and informed consent from interlocutors. All interviews were conducted in the respective church’s premises after observing ethical issues, which included obtaining permission from relevant authorities and the consent of the participants.

## Results

This study aimed to answer the question of whether the religion a person professes plays a crucial role in one’s rejection of the COVID-19 vaccine. Fifty-five interlocutors were interviewed and distributed as follows: 26 from TAG – Makongo-Juu, 16 from Ufufuo na Uzima and 12 from the Arise and Shine Ministries. Out of the interviewed participants, 25 were women and 29 men. Additionally, the average age of the participants was 34 years and ranged from 22 years to 60 years old. In terms of education level, majority of the participants (*n* = 21) had tertiary education, 4 had finished high school education, 19 had ordinary secondary school education and 11 had completed primary education. More details of interlocutors’ demographic information are provided in Table 1. In general, most of the participants (meaning those with secondary to tertiary education) were regarded as educated individuals.

This section presents the findings at three levels based on the socio-ecological models. The findings on the individual and interpersonal relationship focus on sociodemographic variables including gender, age, education level, knowledge, attitude and previous experience of the person concerning vaccines. At the meso level, the focus is on the role of the church, as an institution. The exo-system also covers findings on broad policies and practices that can enable the explanation of uptake of COVID-19 vaccines.

### Religious believers’ attitudes towards COVID-19 vaccines (micro-system/individual factors)

The study findings show that the age of the person was a vital factor in the person’s (un)acceptance of taking the COVID-19 vaccines. Across gender and religion, youths expressed a sense of hesitancy to vaccination than the elderly. In this regard, a participant said:

‘I am not vaccinated because I trust my body’s immune system.’ (P31, 26 years old, male)

This was corroborated by another participant, who similarly affirmed:

‘I am not ready to take the vaccine.’ (P1, 30 years old, female)

She further elaborated that she considered herself less vulnerable as her immune system was strong enough to keep the virus at bay. Implicitly, youths generally perceived vaccines as non-essential, thinking that the virus has no impact on a body endowed with a strong immune system. This perception shaped the youths’ attitude towards COVID-19 vaccines and, hence, affected the uptake. A youth attending a TAG Makongo-Juu Church narrated the preventive mechanisms that people used and stated:

‘Regular exercise and eating natural foods such as fruits and vegetables is a proper means of preventing COVID-19. Those who observe this habit cannot be attacked easily by the virus.’ (P2, 25 years old, male)

As the role of COVID-19 vaccines is to strengthen the body’s immunity to fend off the attack by the virus, such a belief made them dismiss the need for a jab on the account they were covered. Yet, studies have shown that unvaccinated individuals are more likely to spread the virus to others, especially vulnerable groups such as the elderly and people with chronic diseases including human immunodeficiency virus (HIV), heart disease and diabetes than vaccinated ones. As such, the youth’s strong hesitance to COVID-19 vaccines indicated a lack of knowledge of COVID-19 in general and the associated risk they pose to vulnerable social groups.

On the contrary, the findings revealed that the elderly were much more receptive to COVID-19 vaccines. Also, all the elderly people who participated in the study had taken one or two shots of the vaccine at different times and locations. Implicitly, the elderly had a positive attitude towards COVID-19 vaccines. In this regard, a participant exemplifies such an attitude as follows:

‘The COVID-19 vaccine is good because it prevents people from the negative effects of COVID-19 because it boosts our body’s immune system.’ (P3, 50 years old, male)

This positive attitude signals ready awareness and acceptance among the elderly because they are conscious of the risks associated with COVID-19.

When the narratives were analysed across gender, there was no difference in terms of the participants’ attitudes to the COVID-19 vaccine by age. There was consensus that young people were less vulnerable to COVID-19 than elderly people across genders. Nevertheless, the male gender was also perceived to be at greater risk of contracting COVID-19 than their female counterparts. Comparatively, more males had taken COVID-19 vaccines than their female counterparts.

Moreover, almost every participant from Ufufuo na Uzima had a conservative and hardened attitude, mostly a negative one, towards the COVID-19 vaccine. As a result, participants from this congregation attested to being reluctant to take the COVID-19 vaccine. The other two congregations, the Tanzania Assemblies of God and Arise and Shine, on the other hand, seemed to be more liberal and even receptive to taking up the COVID-19 vaccine. Others reported that deciding to accept vaccination was a personal matter that depended on their perception of the severity of the pandemic. In this regard, one participant said:

‘Yes, I am vaccinated! Decisions about vaccination are personal. I thought that prevention was better than cure. Our pastor more often says that you can reject a word but not a calling. As an adult, I understand what is good and what is bad. That prompted me to be vaccinated and it is free of charge. Additionally, the services at the Amana Hospital are very good and if you do not understand, they will clarify [*the matter*] to you.’ (P4, 53 years old, male)

Additionally, the study found that education was not singly an affirmative factor for rejection or acceptance regarding the uptake of the COVID-19 vaccine. The study enrolled participants with a diploma or higher qualification, some of whom persisted with hesitance and a negative attitude to COVID-19 vaccines, implying that education in itself was not a factor that could engender receptivity to the jabs. Some of them accepted COVID-19 and, as a result, had been vaccinated, implying there was another mitigating factor behind such a push.

The study also found that participants who had been infected and/or had COVID-19 victims among relatives, friends or colleagues were more willing to get vaccinated than those without such acquaintances. Indeed, those with previous experience with the impact of COVID-19 on themselves or on other people’s lives were more amenable and expected to get vaccinated than those without such experience.

### Pentecostal church institutional (dis)encouragement on believers

The study findings from participants who congregated at Ufufuo na Uzima, Shine and Arise and Tanzania Assemblies of God Makongo-Juu churches revealed that religion and religious leaders played an important role in the worshippers’ decision-making regarding the uptake of the COVID-19 vaccine. In this regard, the teaching of the church about health-seeking behaviour, preparedness and health risk management emerged to be important and was expected to influence their decisions on such matters. Churches that claimed spiritual healing of COVID-19 had a negative attitude to COVID-19 vaccines, which were treated as an earthly-based cure with less effect than the miracle of spiritual power. A participant from the Ufufuo na Uzima Church affirmed:

‘Prayers are vaccines; there are no vaccines that have more power than those coming from God … Vaccines are useless in the presence of God’s power’. (P5, 38 years old, female)

Another participant said:

‘Satan has brought this disease to test the people’s faith. COVID-19 is propelled by activists who do not believe in God. The correct way to protect oneself from this disease is to pray to God. Tanzanians are perishing because they do not believe in God [*and His protection and healing power*]. Many believe that COVID-19 can be cured without vaccination.’ (P42, 21 years old, male)

The position of the church leader towards the COVID-19 vaccine also emerged to be an important factor in an individual’s decision to reject or accept vaccines. Churches whose leaders dismissed and denounced COVID-19 vaccines biased their followers’ attitudes and practices, often negatively. Specifically, believers from Ufufuo na Uzima affirmed following in the footsteps of their church leaders. A participant from Ufufuo na Uzima Church. said:

‘The teachings of our pastor are very effective in dealing with COVID-19.’ (P14, 38 years old, female)

A believer from TAG Makongo-Juu Church added by saying:

‘At my church, many people attend mass without washing their hands regularly, do not use sanitizers, and do not wear masks. Although our pastor does not wear a mask or wash his hands with water, he encourages people to wash their hands and wear masks. We know the [*COVID-19*] vaccine service is available, but people are not ready to take vaccines. The Pastor encourages people to have physical exercises, and to eat proper diets that make the body fit.’ (P7, 25 years old, male)

Corroboratively, another participant from the same church said:

‘Can’t you see how crowded we are in our Father’s house? … and no one is wearing a mask. God is for the Africans; He protects us; I don’t believe in vaccines.’ (P8, 28 years old, male)

The leader of Ufufuo na Uzima Church, Bishop Gwajima, declared his denunciation position against COVID-19 vaccines. The result of such denunciation was largely negative among the believers and, hence, their reluctance to embrace this protective mechanism.

The participants insinuated that COVID-19 was less devastating to Africans than it was to people of other continents. They attributed it to the favourable position Africans enjoyed to God’s love and intervention for them Africans. As a result, many individuals attempted to justify their hesitance to get vaccinated against COVID-19 by referring to the perceived immunity engendered by the colour of their skin coupled with their location in the tropics with searing heat unpalatable for the survival of the virus, hence presenting them with less at risk or less vulnerable to the ravages of the pandemic.

However, this stance remains contestable in other narratives; in fact, it was contrary to other believers, especially in TAG and Arise and Shine, who presented different positions. For instance, a believer from the Arise and Shine Ministries narrated:

‘It is a disaster for the citizens of Tanzania; it is dangerous and harms the economy, the church’s programmes, business, and other sectors. It can lead to losing faith in where you stand. I believe the vaccine is a preventive mechanism against infection; it prevents getting COVID-19. Although everyone has their own opinion, I do not see any issue with taking a vaccine. The vaccine makes a person confident, even if it is not 100 percent effective, but it removes the fear of being infected.’ (P9, 39 years old, male)

### Religious believers’ attitude towards government policies (macrosystem – ecological and policy) on vaccines

In Tanzania, all religions are governed by laws and policies of the Government of the United Republic of Tanzania. Actually, all regions are supposed to be registered with the Registrar of Societies at the Ministry of Home Affairs. This also means that religious leaders are bound to adhere to government policies regulating public health matters.

Therefore, this study further assumed that the government policy to provide universally cost-free vaccines was a catalyst for increased uptake. Indeed, universal cost-free vaccines, especially for vulnerable groups such as the elderly, were a commendable effort. Such efforts created an impression that the country was considerate of its citizens, hence increasing trust and acceptance, especially among the elderly. In this regard, a participant from Makongo-Juu Church provided an illustrative statement:

‘First of all, I congratulate the government on the effort of providing COVID-19 vaccines because they are provided free-of-charge. This consideration takes cognizance of the economic status of all citizens. COVID-19 is provided free-of-charge and it takes only 30 minutes to get vaccinated. So, everyone should take advantage of the service available at health centres.’ (P10, 50 years old, male)

On the other hand, the hesitant participants questioned the efficacy of the cost-free vaccines. This category of persons had questions revolving around the duration and applicability of the vaccine conditions in Tanzania. Also, the Tanzania government distributed the vaccines to different parts of the country for optimal success, including healthcare centres in villages. As such, citizens could visit any nearby facilities at their convenience. In addition, the government had set up mobile vaccination centres responsive to the needs and events of the respective areas. At TAG Makongo-Juu, for example, the government established a mobile vaccination centre on one of the Sundays for the worshippers to get vaccinated after the mass or during it.

Government policy which restricted travelling without proof of vaccination forced others to vaccinate regardless of their religious inclination. For instance, participants claimed that many of the vaccinated did so because of meeting travel requirements. Because of travel restrictions, only individuals with COVID-19 vaccination proof could travel to countries with restrictions. As a result, some believers became vaccinated not because they believed in the COVID-19 jab as a preventive measure against the virus but because they needed the vaccination certificate to be eligible to travel for business, treatment or official duties.

Consequently, when President Samia Suluhu Hassan took over the presidency in 2021 from the just deceased John Pombe Magufuli, the national atmosphere associated with COVID-19 vaccines and the country changed. The president’s action of making the vaccine a public endeavour increased the people’s receptivity, especially those who had been previously hesitant because of the vaccine’s efficacy and safety. Such action was emulated by religious leaders in their respective churches. In this regard, a participant from TAG Makongo-Juu said:

‘It is a good example for the [*Tanzania*] government to act swiftly. Many government leaders have taken the lead, such as Her Excellency Mama Samia and [*Prof.*] Joyce Ndalichaka [*the Minister of State Employment, Youth and the Disabled*]. The government considers the welfare and well-being of its citizens. By accepting COVID-19 vaccines, the government acted in the best interest of the citizens. However, many citizens are still hesitant about the uptake of the COVID-19 vaccines.’ (P11, 35 years old, female)

Additionally, some participants recalled some of the statements championed by politicians who opposed the COVID-19 vaccinations. Some of the participants remembered the statement by former President Magufuli, a Catholic, and his anti-COVID-19 vaccine stand. Those who supported the President had difficulties in accepting the current COVID-19 vaccines because they trusted his declarations and stand on the virus. For these participants, COVID-19 can be treated using natural remedies readily available in their environment. They said natural remedies such as the daily intake of a mixture of ginger, lemon and garlic and/or *kupiga nyungu* [to steam the body] reduced the impact of the infection. Some religious leaders, including Bishop Gwajima, publicly rejected the COVID-19 vaccines. Regardless of their shortcomings, the speeches from politicians and religious leaders emerged to be significant in shaping the people’s attitude towards COVID-19 vaccines. Some religious leaders, for example, from the Roman Catholic church had cautioned against this kind of positioning against COVID-19 and vaccines in particular as a missional letter addressed to archbishops and retired bishops attested: ‘Our country is not an island. We have every reason to take precautions and pray to God so that we can move unscathed in this pandemic …’^22(p1)^ Consequently, these leaders further showed a positive attitude to vaccines and most of them have since been vaccinated, which encourages their followers to do the same.

## Discussion

The findings of this study have provided evidence at the micro level that sociodemographic variables such as gender, age or history of chronic diseases affected the uptake of COVID-19 vaccines. This also corroborates with extant literature, which established that elderly people are more at risk of being affected than their younger counterparts.^5,7,8,23^ Consequently, the reported death cases associated with COVID-19 showed more of the elderly than other age categories. As a result, the elderly were more inclined to vaccination. Others who were also mentioned as being at risk included people with chronic diseases such as HIV or acquired immunodeficiency syndrome (AIDS). Other elements such as education did not have much impact on the uptake. These findings corroborated other studies, especially in the area of HIV and malaria prevalence, where a high level of knowledge was not affirmative to practice such as the use of condoms and bed nets, respectively.^24^

Moreover, the positions of religious leaders were interconnected to the followers’ perceptions of vulnerability and severity of the virus. In other words, the followers were not passive recipients of the instructions from their church leaders; rather they also cognitively linked to their vulnerable stance and their social environment. Those who were perceived to be at risk of contracting the virus, such as the elderly, did not heed the instructions of the religious leaders as opposed to the youth and women, who strongly believed they could fend off the disease with their immunity.

The above arguments showed mixed feelings: on one hand, religions attributed to believers’ attitude towards the vaccine, and on the other hand, other institutions such as the place of work also contributed to believers’ decisions towards the vaccines. This corroborates with the socio-ecological model, which suggests that institutions are important in the persons’ decision making and play a key role in imparting health values and reciprocity.^8^ This study signified that indeed institutions are strong and regulate behaviours and choices including health-seeking behaviour, which in this case acceptance of COVID-19 vaccines. Nevertheless, participants’ narratives showed that individuals engaged in a cognitive process before accepting COVID-19 vaccines as expressed by Van Dijk.^25^ In other words, individuals assessed the benefits and risks of taking the jab.

Furthermore, religion amplified the significance of the social-ecological framework. In this regard, the believers who were inclined by religious teaching and utterances of their religious leaders were expected to provide inspiration to their peers, family members and co-workers; vice versa was true. Furthermore, as the utterances of prominent religious leaders such as Gwajima – the Bishop and founder of the Ufufuo na Uzima Ministry were in the public domain and on social media, their effects transcended the confines of the believers of the respective churches. Other people’s decisions on COVID-19 vaccines were also influenced by the anti-vaccine utterances. Overall, rejection and religious objections to COVID-19 vaccines were attributable to attitudes to the COVID-19 vaccines grounded not only in religious teachings but also in many aspects of the social-ecological framework, hence making the effects multi-faceted. In addition to the socio-ecological framework are the aspects of cognition and agency. That means individuals do not only react to stimuli from the surroundings but also use their cognitive process and navigate their decisions as active agents of the contextual discourse.

Findings regarding people’s attitude towards government policies on COVID-19 vaccination have provided a picture of a responsible government. The narrations on government policies that provided free COVID-19 vaccines for all individuals and covering the entire country exemplified such attitude. Immediate government response to protect people who were at risk such as the elderly and those who have chronic diseases cemented people’s positive attitude towards government policies. Nevertheless, the implication of this positive attitude on people’s practice can be attributed to the socio-ecological factors that the individual was exposed to. This was reflected on people’s readiness to accept or reject the uptake of the vaccine.

## Conclusion

The findings presented in the study suggest that religion alone cannot be the sole basis of either acceptance or rejection of COVID-19 vaccines. Other factors at play (e.g., age, gender, work and the social environment, just to mention a few) coupled with religious factors affected the decision of Pentecostal believers to either accept or reject COVID-19 vaccines. Nevertheless, religion and religious agents or institutions played a dominant role in the lives of Tanzanians.

Furthermore, different government policies and directives that were formulated to guide COVID-19 vaccination were received with mixed attitude. The individuals supported policies that ensured the provision of free COVID-19 vaccines to the general population but were against policies that would force every citizen to have COVID-19 vaccines. The findings indicated that the voluntary nature of government policies increased acceptability and removed fear among the people who wanted to have the jab.

Indeed, Pentecostal believers who alleged were invulnerable, such as the youth and women, tended to mimic the behaviour and attitude of their religious leaders to show allegiance and solidarity with them. Additionally, the church and/or religious leaders who emphasised the spiritual power of healing implied that anyone who opted for vaccination demeaned the power of the divine intervention and, hence, had a weak belief and was spiritually immature. In this situation, statements like ‘We trust our God’, ‘in God we trust’ and ‘We can survive through prayers and belief in God’ were common. This kind of attitude and utterances were expected to come from the youth and women whose turnout at vaccination centres was generally low. Nevertheless, public health has to consider these multi-faceted and inter-sectionality dimensions of health interventions, including religion as an important factor in influencing people’s attitudes and practices.
